# Reply to Alvarez and Llibre

**DOI:** 10.1093/ofid/ofad669

**Published:** 2024-01-11

**Authors:** David P Serota, Maria G Rodriguez, Alina Syros, Allan E Rodriguez

**Affiliations:** Division of Infectious Diseases, Department of Medicine, University of Miami Miller School of Medicine, Miami, Florida, USA; Division of Infectious Diseases, Department of Medicine, University of Miami Miller School of Medicine, Miami, Florida, USA; Division of Infectious Diseases, Department of Medicine, University of Miami Miller School of Medicine, Miami, Florida, USA

To the Editor—We appreciate Alvarez and Llibre's commentary on our viewpoint and appreciate them bringing to our attention the articles by Ding et al [1] and Elvstam et al [2], which both report a statistically significant association between viral loads (VLs) 50–200 copies/mL and the clinical consequences of all-cause mortality and non-AIDS events, respectively. Despite these studies, we maintain our conclusions that the clinical relevance of VLs between the lower limit of detection and 200 copies/mL (low-level viremia [LLV]) remains inconclusive. Thus, it is not certain that the benefits of reporting such values, especially to patients, outweigh the potential harms.

The authors provide a helpful table with multiple cohorts evaluating the association of detectable VLs <200 copies/mL and clinical outcomes. Of the presented data, only 2 of 8 hazard ratios show statistical significance. Six of eight show no significant association. We question the results of Elvstam et al because they hypothesize that LLV causes increased mortality yet find no association with increased AIDS events, non-AIDS events, or cardiovascular events [2, 3]. The largest discrepancy in causes of death between the virally suppressed patients and detectable VL <200 copies/mL appears to be increased accidental/violent death among those with LLV. We suspect this could be a spurious or confounded finding. The study by Ding et al includes only 53 participants with VL 50–199 copies/mL and 8 non-AIDS events, so we hesitate to apply this result to such a large potential population when larger studies show no association [1]. We believe the cited findings of biomarker studies are certainly of concern but would consider them hypothesis-generating. There is also a question of causality of detectable human immunodeficiency virus (HIV) VL on these negative outcomes. Is it the low levels of viral particles/RNA in the blood directly leading to inflammation, death, or non-AIDS events? Or is LLV a sign of another causal factor, such as high viral reservoir, or other viral/host factors [4]?

Regarding the authors’ suggested actions to take when facing detectable HIV RNA <200 copies/mL, we do not believe these items are proven to improve the care or clinical outcomes of this specific patient population, though they all seem like reasonable interventions that could be universally employed. The primary goal of our manuscript was to bring attention to the frequently encountered problem of detectable VLs <200 copies/mL and the potential harms of how these low values are used—or misused—in clinical practice. Moreover, we sought to emphasize how reporting of detectable VLs <200 copies/mL could cause further harm by confusing trusted public health initiatives such as “U = U,” a concept that has revolutionized the care of people with HIV. Suppressed reporting of low VLs is one potential solution, though certainly has potential downsides and may not be the correct fix. We are supportive of further research into causes, implications, and interventions for LLV, but maintain that in the present day there remains little compelling evidence that knowledge about detectable VLs <200 copies/mL should play a role in routine clinical care of individuals with HIV.

## Note

***Potential conflicts of interest*.** All authors: No reported conflicts.
